# Supplementary figures and images for: Developmental demands contribute to early neuromuscular degeneration in CMT2D mice
Source: Cell Death Dis. 2020 Jul 23;11(7):564. doi: 10.1038/s41419-020-02798-y (PMC7378196; doi:10.1038/s41419-020-02798-y)

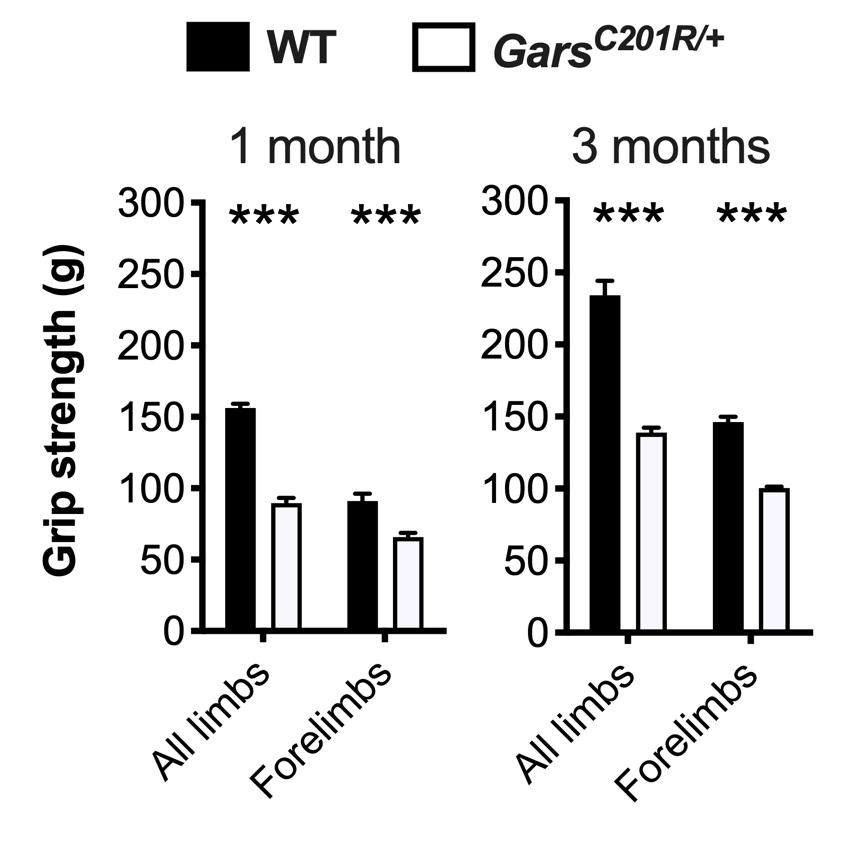

Supplement: Supplementary file 1 — Supplementary Figure S1 [file 41419_2020_2798_MOESM1_ESM.tif]

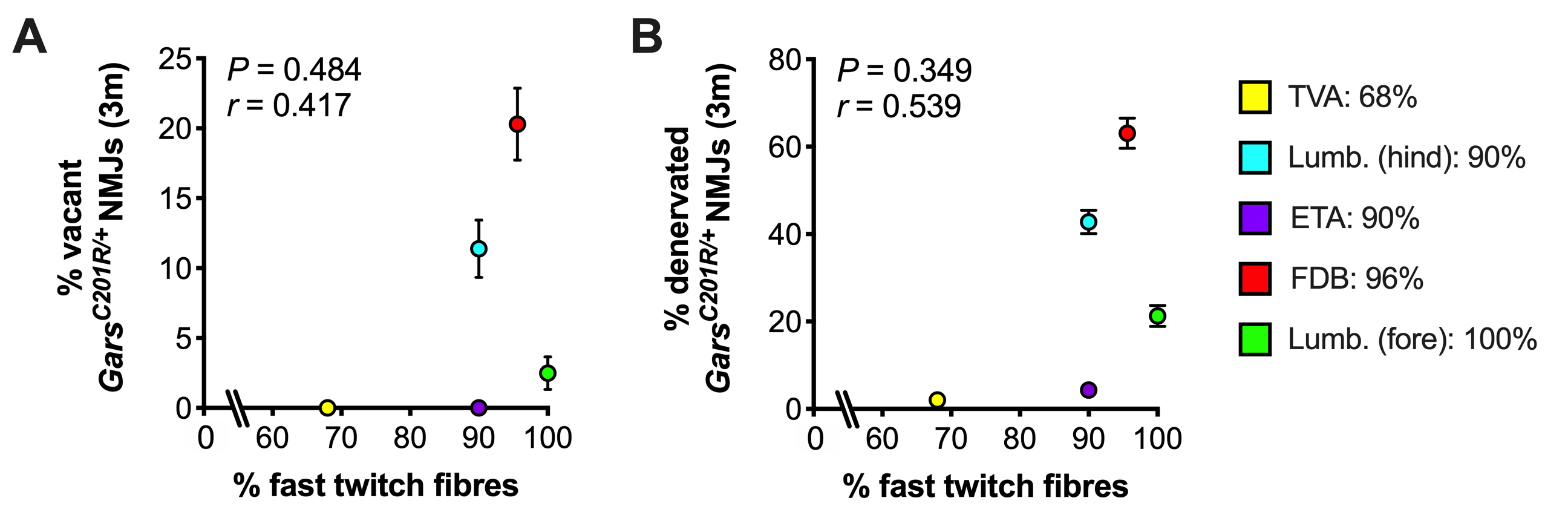

Supplement: Supplementary file 2 — Supplementary Figure S2 [file 41419_2020_2798_MOESM2_ESM.tif]

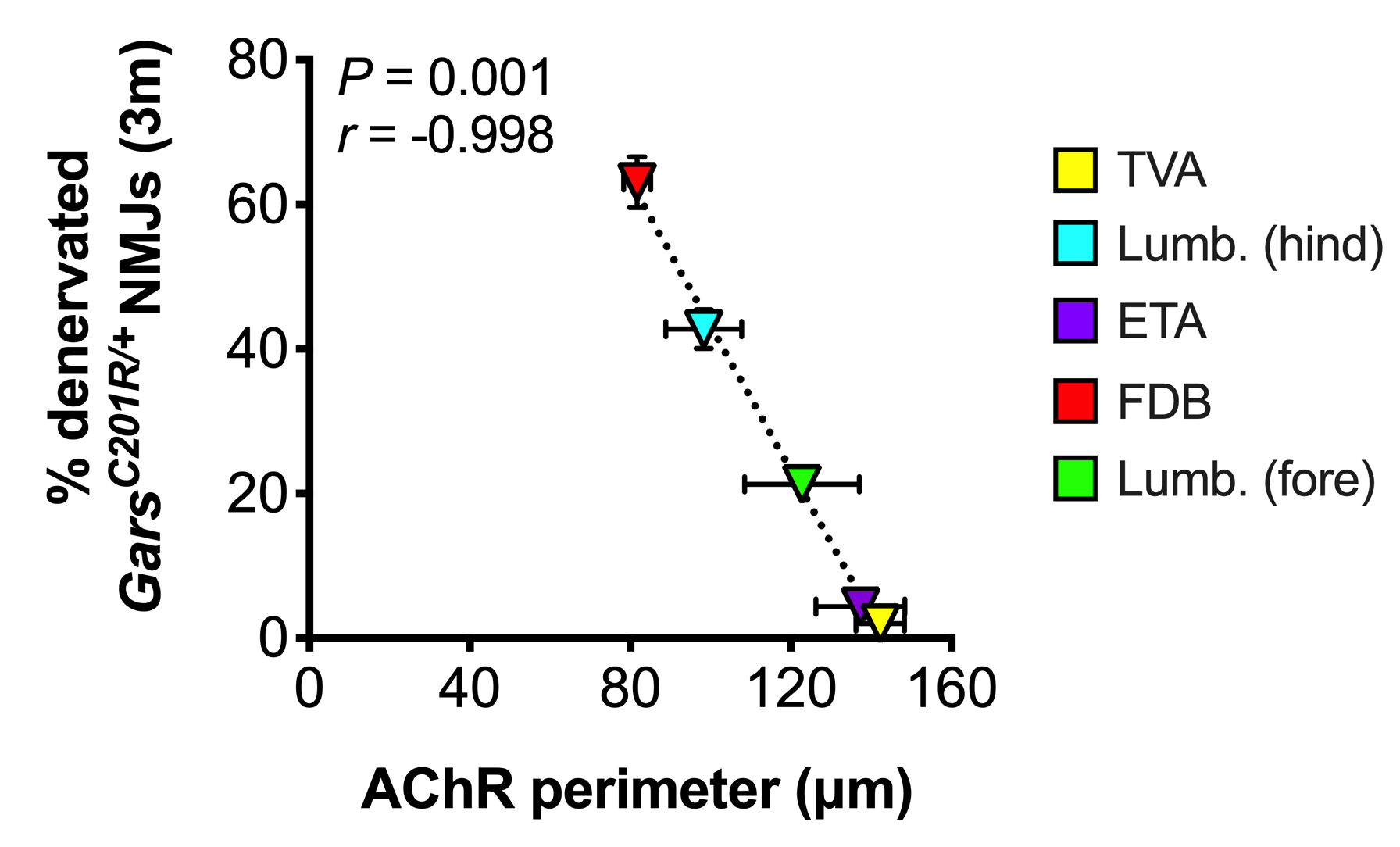

Supplement: Supplementary file 3 — Supplementary Figure S3 [file 41419_2020_2798_MOESM3_ESM.tif]
