# Supplementary material for: Developmental demands contribute to early neuromuscular degeneration in CMT2D mice
Source: Cell Death Dis. 2020 Jul 23;11(7):564. doi: 10.1038/s41419-020-02798-y (PMC7378196; doi:10.1038/s41419-020-02798-y)
Supplement: Supplementary file 4 — Supplementary Information [file 41419_2020_2798_MOESM4_ESM.docx]

**Supplementary Figure Legends**

**Supplementary Figure S1. Raw grip strength data.** Grip strength of all limbs and just the forelimbs are both significantly impaired in *Gars^C201R/+^* mice at one (left, genotype *P* < 0.001, limbs *P* < 0.001, interaction *P* < 0.001; two-way ANOVA) and three months (right, genotype *P* < 0.001, limbs *P* < 0.001, interaction *P* < 0.001; two-way ANOVA). *N.b.*, only females were analysed, “All limbs” data has been previously published^1^, and different mice were used for the two timepoints and for testing all limbs vs. forelimbs. *n* = 4 (*Gars^C201R/+^* forelimbs at three months), 6 (one month and all limbs at three months) and 8 (wild-type forelimbs at three months). Means ±SEM are plotted. ^***^*P* < 0.001; Sidak's multiple comparisons test. See also **Fig. 1**.

**Supplementary Figure S2. CMT2D denervation does not correlate with muscle fibre type.** (**A**, **B**) There is no correlation between fast twitch fibres percentage found in each muscle (see legend for %) and percentage of vacant (A, *P* = 0.484, *r* = 0.417, Pearson’s product moment correlation) or denervated (B, *P* = 0.349, *r* = 0.539, Pearson’s product moment correlation) NMJs at three months in *Gars^C201R/+^* mice. *n* = 6. Means ±SEM are plotted for % vacancy (A) and % denervation (B) in CMT2D mice. *Lumb. (fore)*, forepaw lumbricals; *Lumb. (hind)*, hindpaw lumbricals. See also **Fig. 4**.

**Supplementary Figure S3. AChR perimeter of immature NMJs is the only morphological variable to correlate with CMT2D denervation.** Correlation was assessed between the percentage of vacant or denervated NMJs in three month old *Gars^C201R/+^* mice and 21 different NMJ morphological variables from wild-type mice at both P7 and P31-32. Wild-type morphological data^2^ and TVA vacancy/denervation data^3^ have been published elsewhere. Correlation was assessed by calculating Pearson’s product moment correlation coefficient (*r*), the results of which are presented in **Supplementary Table S2** (% vacant) and **Supplementary Table S3** (% denervated) along with associated *P* values. Of 82 different tests, only AChR perimeter at P7 significantly correlated (Bonferroni-corrected *P* < 0.00244 for the 21 tests per timepoint) with the percentage denervation at the CMT2D NMJ (*P* = 0.001, *r* = -0.998). *n* = 6 (all muscles except TVA) and 8 (TVA). Means ±SEM are plotted. *Lumb. (fore)*, forepaw lumbricals; *Lumb. (hind)*, hindpaw lumbricals.

**Supplementary Tables**

|  | | **TVA vs. Lumb. (hind)** | **TVA vs. ETA** | **TVA vs. FDB** | **TVA vs Lumb. (fore)** | **Lumb. (hind) vs. ETA** | **Lumb. (hind) vs. FDB** | **Lumb. (hind) vs Lumb. (fore)** | **ETA vs. FDB** | **ETA vs. Lumb. (fore)** | **FDB vs. Lumb. (fore)** |
| --- | --- | --- | --- | --- | --- | --- | --- | --- | --- | --- | --- |
| **% NMJs** | **ANOVA *P* value** |  |  |  |  |  |  |  |  |  |  |
| Fully innervated | **<0.001** | ******* | *ns* | ******* | ******* | ******* | ******* | ******* | ******* | ******* | ******* |
| Partially denervated | **<0.001** | ******* | *ns* | ******* | ******* | ******* | ******* | ******* | ******* | ******* | ******* |
| Vacant | **<0.001** | ******* | *ns* | ******* | *ns* | ******* | ****** | ****** | ******* | *ns* | ******* |
| Polyinnervated | **<0.001** | ***** | *ns* | ******* | *ns* | *ns* | ***** | *ns* | ******* | *ns* | ******* |

**Supplementary Table S1. Pairwise significance testing between CMT2D mouse muscle denervation and polyinnervation. ***** *P* < 0.001, ** *P* < 0.01, * *P* < 0.05, *ns* not significant; Tukey's multiple comparisons test. *Lumb. (fore)*, forepaw lumbricals; *Lumb. (hind)*, hindpaw lumbricals. See also **Fig. 4**.

|  | **P7** | | **P31-32** | |
| --- | --- | --- | --- | --- |
| **Variable** | ***r*** | **Pearson *P* value** | ***r*** | **Pearson *P* value** |
| Polyinnervation (%) | -0.160 | 0.800 | -0.538 | 0.350 |
| Nerve terminal perimeter (µm) | -0.896 | 0.040 | -0.499 | 0.393 |
| Nerve terminal area (µm^2^) | -0.932 | 0.021 | -0.447 | 0.451 |
| # terminal branches | -0.769 | 0.128 | -0.490 | 0.404 |
| # branch points | -0.825 | 0.086 | -0.194 | 0.767 |
| Total branch length (µm) | -0.974 | 0.053 | -0.463 | 0.433 |
| Average branch length (µm) | -0.137 | 0.826 | 0.038 | 0.951 |
| Complexity | -0.883 | 0.047 | -0.294 | 0.632 |
| Axon diameter (µm) | -0.757 | 0.139 | -0.563 | 0.334 |
| AChR perimeter (µm) | -0.978 | 0.004 | -0.451 | 0.446 |
| AChR area (µm^2^) | -0.884 | 0.046 | -0.427 | 0.473 |
| Endplate diameter (µm) | -0.718 | 0.172 | -0.362 | 0.550 |
| Endplate perimeter (µm) | -0.840 | 0.075 | -0.447 | 0.451 |
| Endplate area (µm^2^) | -0.900 | 0.038 | -0.498 | 0.393 |
| # AChR clusters | -0.930 | 0.022 | -0.613 | 0.272 |
| AChR cluster area (µm^2^) | -0.809 | 0.097 | 0.846 | 0.071 |
| Compactness (%) | -0.915 | 0.029 | 0.730 | 0.162 |
| Fragmentation | -0.990 | 0.038 | -0.652 | 0.233 |
| Muscle fibre diameter (µm) | *n/a* | *n/a* | -0.441 | 0.457 |
| Synaptic contact area (µm^2^) | -0.920 | 0.027 | -0.450 | 0.447 |
| Overlap (%) | -0.889 | 0.044 | 0.216 | 0.728 |

**Supplementary Table S2. Statistical testing of correlation between the percentage of vacant *Gars^C201R/+^* NMJs at three months and wild-type NMJ morphological variables at P7 and P31-32.** Pre-synaptic variables are shaded green, post-synaptic variables shaded purple, and combined pre- and post-synaptic variables are unshaded. *n/a*, not applicable as wild-type morphological data not available at P7. See also **Supplementary** **Fig. S3**.

|  | **P7** | | **P31-32** | |
| --- | --- | --- | --- | --- |
| **Variable** | ***r*** | **Pearson *P* value** | ***r*** | **Pearson *P* value** |
| Polyinnervation (%) | -0.214 | 0.730 | -0.611 | 0.274 |
| Nerve terminal perimeter (µm) | -0.963 | 0.009 | -0.641 | 0.244 |
| Nerve terminal area (µm^2^) | -0.982 | 0.003 | -0.573 | 0.313 |
| # terminal branches | -0.873 | 0.053 | -0.641 | 0.244 |
| # branch points | -0.915 | 0.029 | -0.281 | 0.647 |
| Total branch length (µm) | -0.949 | 0.014 | -0.603 | 0.282 |
| Average branch length (µm) | 0.017 | 0.978 | 0.221 | 0.721 |
| Complexity | -0.956 | 0.011 | -0.447 | 0.450 |
| Axon diameter (µm) | -0.647 | 0.238 | -0.604 | 0.281 |
| AChR perimeter (µm) | -0.998 | **0.001^#^** | -0.601 | 0.283 |
| AChR area (µm^2^) | -0.956 | 0.011 | -0.571 | 0.315 |
| Endplate diameter (µm) | -0.814 | 0.093 | -0.491 | 0.401 |
| Endplate perimeter (µm) | -0.922 | 0.026 | -0.687 | 0.299 |
| Endplate area (µm^2^) | -0.964 | 0.008 | -0.639 | 0.246 |
| # AChR clusters | -0.864 | 0.059 | -0.745 | 0.149 |
| AChR cluster area (µm^2^) | -0.903 | 0.036 | 0.901 | 0.037 |
| Compactness (%) | -0.973 | 0.005 | 0.845 | 0.071 |
| Fragmentation | -0.844 | 0.072 | -0.778 | 0.121 |
| Muscle fibre diameter (µm) | *n/a* | *n/a* | -0.571 | 0.315 |
| Synaptic contact area (µm^2^) | -0.978 | 0.004 | -0.567 | 0.319 |
| Overlap (%) | -0.819 | 0.090 | 0.383 | 0.524 |

**Supplementary Table S3. Statistical testing of correlation between the percentage of denervated *Gars^C201R/+^* NMJs at three months and wild-type NMJ morphological variables at P7 and P31-32.** Pre-synaptic variables are shaded green, post-synaptic variables shaded purple, and combined pre- and post-synaptic variables are unshaded. **^#^***P* < 0.00244 Pearson’s product moment correlation, *i.e.* the Bonferroni correction-adjusted *P* value for an α of 0.05 when performing 21 associated tests. *n/a*, not applicable as wild-type morphological data not available at P7. See also **Supplementary** **Fig. S3**.

|  | **P7 to P31-32** | |
| --- | --- | --- |
| **Variable** | ***r*** | **Pearson *P* value** |
| Polyinnervation (%) | -0.257 | 0.677 |
| Nerve terminal perimeter (µm) | 0.968 | 0.007 |
| Nerve terminal area (µm^2^) | 0.986 | **0.002^#^** |
| # terminal branches | 0.990 | **0.001^#^** |
| # branch points | 0.964 | 0.008 |
| Total branch length (µm) | 0.993 | **<0.001^#^** |
| Average branch length (µm) | 0.289 | 0.637 |
| Complexity | 0.982 | 0.003 |
| Axon diameter (µm) | -0.069 | 0.912 |
| AChR perimeter (µm) | 0.643 | 0.242 |
| AChR area (µm^2^) | 0.994 | **<0.001^#^** |
| Endplate diameter (µm) | 0.956 | 0.011 |
| Endplate perimeter (µm) | 0.943 | 0.016 |
| Endplate area (µm^2^) | 0.955 | 0.012 |
| # AChR clusters | -0.215 | 0.728 |
| AChR cluster area (µm^2^) | 0.928 | 0.023 |
| Compactness (%) | 0.882 | 0.048 |
| Fragmentation | -0.398 | 0.507 |
| Synaptic contact area (µm^2^) | 0.995 | **<0.001^#^** |
| Overlap (%) | 0.833 | 0.080 |

**Supplementary Table S4. Statistical testing of correlation between the percentage of vacant *Gars^C201R/+^* NMJs at three months and the percentage change in wild-type NMJ morphological variables from P7 to P31-32.** Pre-synaptic variables are shaded green, post-synaptic variables shaded purple, and combined pre- and post-synaptic variables are unshaded. **^#^***P* < 0.00256 Pearson’s product moment correlation, *i.e.* the Bonferroni correction-adjusted *P* value for an α of 0.05 when performing 20 associated tests. See also **Fig. 5**.

|  | **P7 to P31-32** | |
| --- | --- | --- |
| **Variable** | ***r*** | **Pearson *P* value** |
| Polyinnervation (%) | -0.207 | 0.738 |
| Nerve terminal perimeter (µm) | 0.919 | 0.028 |
| Nerve terminal area (µm^2^) | 0.962 | 0.009 |
| # terminal branches | 0.962 | 0.009 |
| # branch points | 0.988 | **0.002^#^** |
| Total branch length (µm) | 0.977 | 0.004 |
| Average branch length (µm) | 0.460 | 0.435 |
| Complexity | 0.986 | **0.002^#^** |
| Axon diameter (µm) | -0.190 | 0.760 |
| AChR perimeter (µm) | 0.496 | 0.395 |
| AChR area (µm^2^) | 0.968 | 0.007 |
| Endplate diameter (µm) | 0.948 | 0.014 |
| Endplate perimeter (µm) | 0.896 | 0.040 |
| Endplate area (µm^2^) | 0.896 | 0.040 |
| # AChR clusters | -0.388 | 0.519 |
| AChR cluster area (µm^2^) | 0.980 | 0.003 |
| Compactness (%) | 0.954 | 0.012 |
| Fragmentation | -0.554 | 0.332 |
| Synaptic contact area (µm^2^) | 0.979 | 0.004 |
| Overlap (%) | 0.883 | 0.047 |

**Supplementary Table S5. Statistical testing of correlation between the percentage of denervated *Gars^C201R/+^* NMJs at three months and the percentage change in wild-type NMJ morphological variables from P7 to P31-32.** Pre-synaptic variables are shaded green, post-synaptic variables shaded purple, and combined pre- and post-synaptic variables are unshaded. **^#^***P* < 0.00256 Pearson’s product moment correlation, *i.e.* the Bonferroni correction-adjusted *P* value for an α of 0.05 when performing 20 associated tests. See also **Fig. 5**.

**Supplementary References**

1. Sleigh, J. N. *et al.* Trk receptor signaling and sensory neuron fate are perturbed in human neuropathy caused by *Gars* mutations. *Proc. Natl. Acad. Sci. USA* **114**, E3324–E3333 (2017).

2. Mech, A. M., Brown, A. L., Schiavo, G. & Sleigh, J. N. Morphological variability is greater at developing than mature mouse neuromuscular junctions. *J. Anat.* in press (2020) doi: 10.1111/joa.13228.

3. Sleigh, J. N., Grice, S. J., Burgess, R. W., Talbot, K. & Cader, M. Z. Neuromuscular junction maturation defects precede impaired lower motor neuron connectivity in Charcot-Marie-Tooth type 2D mice. *Hum. Mol. Genet.* **23**, 2639–2650 (2014).
